# Peripheral blood immunoprofiling in patients with polypropylene mesh implants for hernia repair: a single-center cohort study

**DOI:** 10.1007/s10029-025-03310-1

**Published:** 2025-04-01

**Authors:** Barbora Jíšová, Matyáš Ebel, Andrew de Beaux, Barbora East

**Affiliations:** 1 3rd Department of Surgery, First Faculty of Medicine, Charles University and Motol University Hospital, Prague, Czech Republic; 2 Department of Paediatric Neurology, Second Faculty of Medicine, Charles University and Motol University Hospital, Prague, Czech Republic; 3 Department of Surgery, Royal Infirmary of Edinburgh, Edinburgh, UK

**Keywords:** Hernia repair, Polypropylene mesh, Autoimmunity, Immune reaction, Surgical mesh, Immunoglobulin G, ANCA, Rheumatoid factor

## Abstract

**Purpose:**

Surgical mesh, often made from polypropylene, is commonly recommended to enhance hernia repair outcomes in adults. Concerns about polypropylene, as a cause of allergy and/or autoimmune disease prompted this study to evaluate immunological parameters in patients with mesh and healthy controls.

**Methodology:**

A case-control cohort study was conducted at a university hospital. Electronic patient records of hernia repairs using polypropylene mesh (January 2018-April 2022) were analysed. Blood samples from patients and healthy controls were assessed using various methods, including enzyme-linked immunosorbent assay (ELISA), immunofluorescence, immunoblotting, and flow cytometry.

**Results:**

The database search identified 1544 participants. After applying the exclusion criteria 33 patients remained in the polypropylene mesh group. Patients with mesh had lower median IgG3 levels (*p* = 0.02) and Rheumatoid factor (RF) IgM (*p* = 0.018) compared to the control group. Although both IgG3 and RF IgM levels were in the normal reference range. In addition, 5 patients in the mesh group tested positive for serum ANCA levels compared to none in the control group (*p* = 0.053). No other differences in immunoglobulins, autoantibodies, complement, or immune cell subtypes were observed.

**Conclusion:**

Patients with polypropylene mesh exhibited median IgG3 and RF IgM serum levels that were within the normal reference range but slightly lower compared to the control group. Among patients with polypropylene mesh, five displayed positive serum ANCA levels without autoimmune-related symptoms. Overall, no definitive signs of autoimmunity caused by polypropylene mesh. A larger, prospective study is warranted to further explore potential immune responses to polypropylene mesh.

**Supplementary information:**

The online version contains supplementary material available at 10.1007/s10029-025-03310-1.

## Introduction

The use of surgical mesh has been shown to significantly reduce the recurrence of hernias after repair. The European Hernia Society guidelines recommend the use of mesh in the repair of hernias in many localisations [1, 2]. However, there is debate about side effects and autoimmune reactions following implantation of foreign materials. Autoimmune/Inflammatory Syndrome Induced by Adjuvants (ASIA syndrome) is characterised by the development of autoimmune or inflammatory manifestations following exposure to certain adjuvants[3]. Although the exact mechanisms underlying ASIA syndrome remain incompletely understood, several theories have been proposed. These include immune dysregulation, molecular mimicry, chronic stimulation, genetic susceptibility and environmental factors[4]. In 2022, a comprehensive review of the pertinent literature regarding autoimmune reactions following hernia polypropylene mesh implantation was conducted. The analysis failed to demonstrate any correlation between the use of surgical mesh in hernia surgery and subsequent autoimmune reactions, although the study acknowledged the very low quality of published data[5].

It has been posited that the exposure to adjuvants potentially triggers a perturbation in immune homeostasis, thereby engendering the initiation of immune responses directed towards self-tissues. Such dysregulation encompasses a spectrum of immune constituents, comprising but not limited to T cells, B cells, and cytokines[4]. Besides well-known adjuvants such as aluminium hydroxide or silica, polypropylene mesh has been suggested to serve as an adjuvant although this has never been confirmed[5].

To bridge this knowledge gap about human body reaction to polypropylene, our study analysed immune parameters in patients several years post polypropylene mesh implantation comparing them to healthy controls, aiming to uncover potential disparities in their immunological profiles.

## Methodology

### Study design and population

The inclusion criteria for the study group were defined as a history of a hernia repair at least 1 year previously using a polypropylene mesh implant (between January 2018 and April 2022).

The Motol University Hospital database of patients treated at the 3rd Department of Surgery was interrogated and electronic patient records were searched to identify study participants. We identified 1,544 patients who underwent hernia repair. The exclusion criteria were set as age under 18 or over 60 years. In addition, based on patients´ electronic records, those who had a different type of mesh, had a personal history of any form of autoimmune disease prior to the hernia repair, had allergy or malignancy and any form of postoperative complication higher than CD score I, or hernia recurrence, were excluded. This process identified 130 subjects. Some declined to participate, while others lacked a listed phone number in the databases and were not contactable. Ultimately, 33 patients were included in the mesh group. (Diagram 1).

The control group consisted of case-matched healthy individuals with no history of autoimmune disease, allergy, cancer, hernia or any foreign material implant. Participants for the control group were recruited through social media and screened in a similar manner to the enrolled patients.

Clinical datasets were anonymised and all identifiable and traceable links to an individual were removed. All recipients provided written informed consent to approve the use of their medical data for this research. The study adhered to the principles of the Declaration of Helsinki and was approved by the Ethics Committee of Motol University Hospital (EK-361/23).

### Laboratory methods

Peripheral venous blood samples were collected into vacuum tubes and processed according to standardised protocols. The blood samples were processed immediately after blood collection. Enzyme-linked immunosorbent assay (ELISA) methods were employed for quantification of IgG, IgA, IgM, IgE, C3, C4, CIK, C1Q, and CRP levels. Autoantibodies (ANA, ANCA, dsDNA, RF) were detected utilising immunofluorescence or immunoblotting assays. Flow cytometry techniques were utilised for the enumeration of various cellular subsets including Leukocytes, Lymphocytes, CD3+, CD4+, and CD8 + T cells, NK cells (CD3-CD16 + CD56+), B cells (CD19+), and the calculation of CD4+/CD8 + ratios.

Quality control measures were implemented throughout the experimental procedures to ensure accuracy and reliability of the obtained data.

### Statistical analyses

Baseline characteristics were summarised using descriptive statistics. For categorical variables, we employed Fisher’s exact test to assess associations between immunological markers and patient groups. This test was selected to ensure accuracy with smaller sample sizes.

Continuous variables were reported as a median (min-max). Their normality was first assessed using the one-sample Kolmogorov-Smirnov test. In cases where the data were normally distributed, we used a one-way ANOVA test to compare values across patient groups; otherwise, the Kruskal-Wallis test was employed. Data visualisation included heatmaps for categorical data and boxplots for continuous data, with significance levels annotated on the plots. Each boxplot represents the interquartile range (IQR), with the red line denoting the median. ‘Whiskers’ extend to values within 1.5 times the IQR from the quartiles, while points beyond are marked as outliers. *P*-values from individual tests are annotated on both heatmaps and boxplots.

Statistical significance was defined as *p*-values < 0.05. All analyses were conducted using MATLAB R2021b.

## Results

The hospital database included 1,544 consecutive patients who underwent repair of groin, primary ventral, or incisional hernias. Of these, 1,024 patients were older than 60 years, 132 patients underwent a non-mesh repair, and 67 received a non-polypropylene type of mesh. Patients with a history of allergies (*n* = 184), autoimmune diseases or cancer (*n* = 74) were excluded. In addition, 86 patients either had no phone number listed in their medical records or did not respond to phone calls. Forty-four patients were invited to come for clinical examination and a blood test but 11 did not attend their appointment. Thus 33 patients (26 males) were included in the study (Table 1). The median time since hernia repair surgery was 48.5 (12–70) months at the time of blood sampling.

**Table 1:** Demographic characteristics of patients in the mesh and control groups

	Group		
	Mesh	Control	*P*-value
Number of participans	33	31	x
Median of age #	48.0 (11.0)	46.0 (7.0)	x
BMI #	26.8 (4.8)	24.0 (4.4)	x
Month after surgery #	48.5 (11.0)	x	x
Female	7	6	x
Male	26	25	x
Lichtenstein/cica/ umbilical	24 / 7 / 2	x	x
IgG			0.616
IgG1			0.167
IgG2			0.288
IgG3			0.002
RF IgM			0.018
ANCA			0.053

# data represented as median (interquartile range)

The control group consisted of 31 patients (25 males) aged between 18 and 60 years (median age of 46 (23–59) years) without any allergies, autoimmune diseases, or cancer in their personal history and no surgical implants including polypropylene mesh (Table 1), of similar age who were willing to participate in the study.

**Fig. 1:**
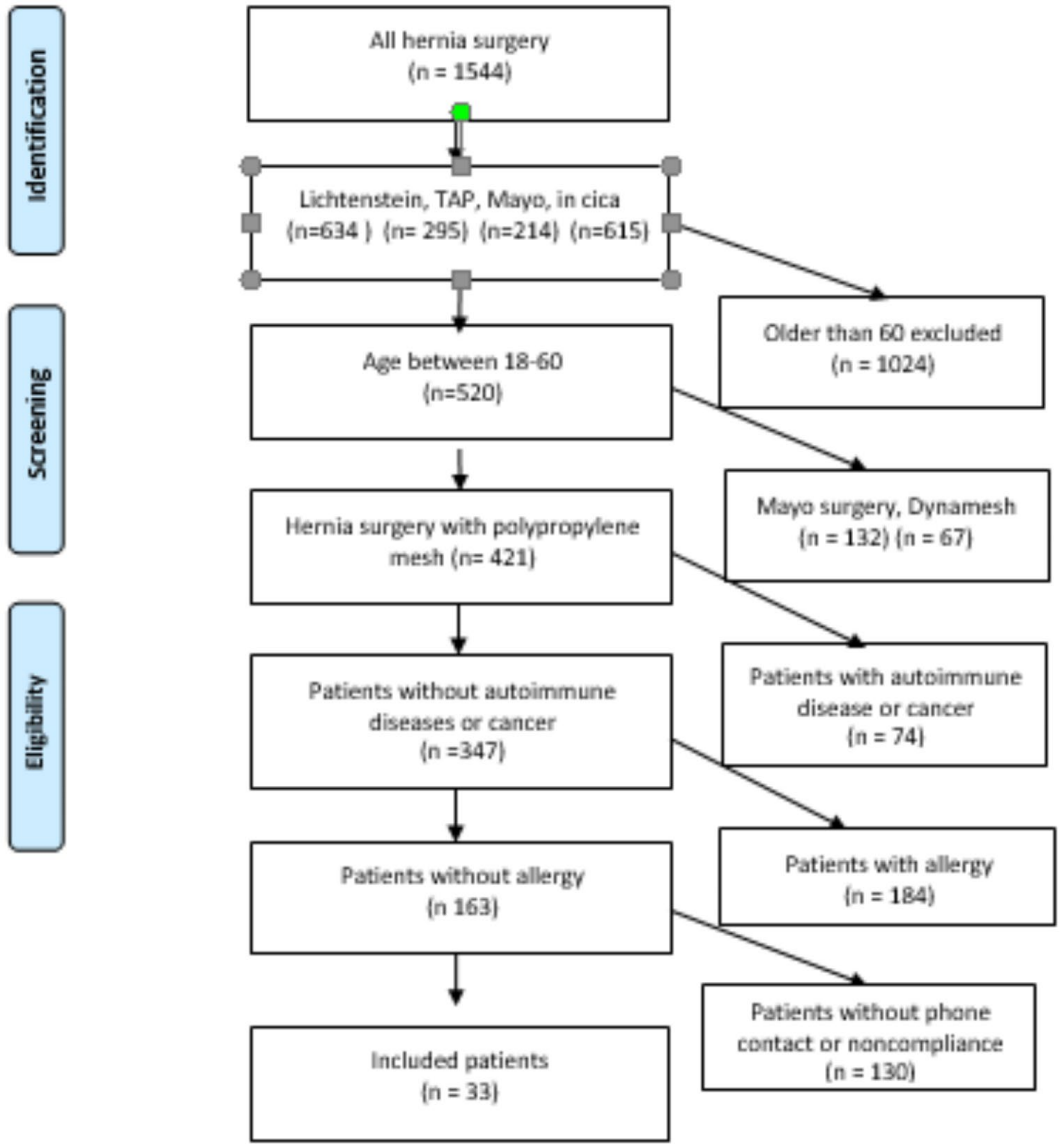
Flow diagram of patients selection for cohort study

All blood tests were performed between June and December 2023. The levels of the immunoglobulin classes (IgG, IgA, IgM, IgE) were analysed by ELISA with no statistically significant differences between the groups (IgG: *p* = 0.616, IgA: *p* = 0.374, IgM: *p* = 0.752, IgE: *p* = 0.216) Fig. 1.

**Fig. 2:**
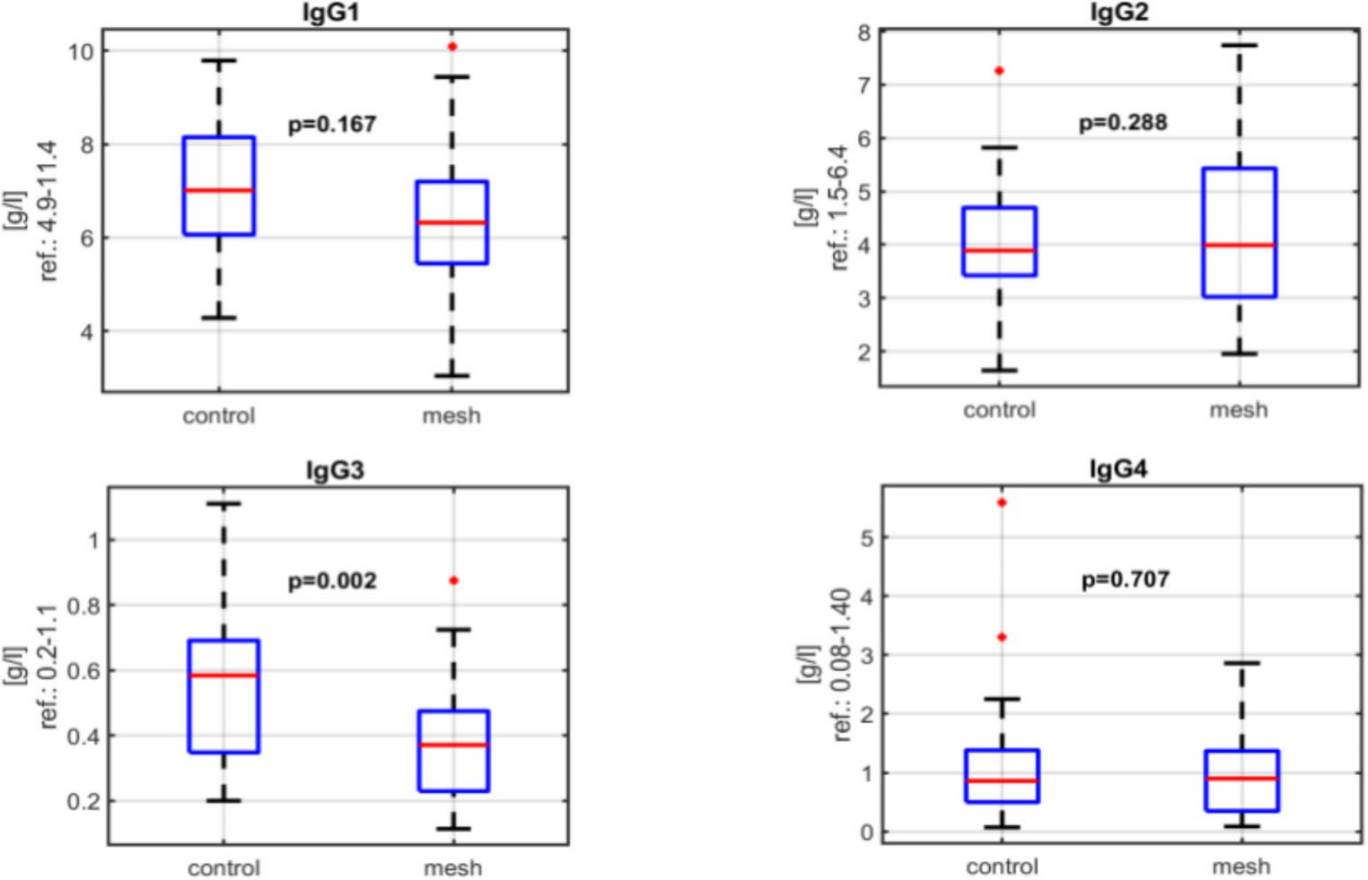
IgG levels amongst patients in the mesh and control groups

**Fig. 3:**
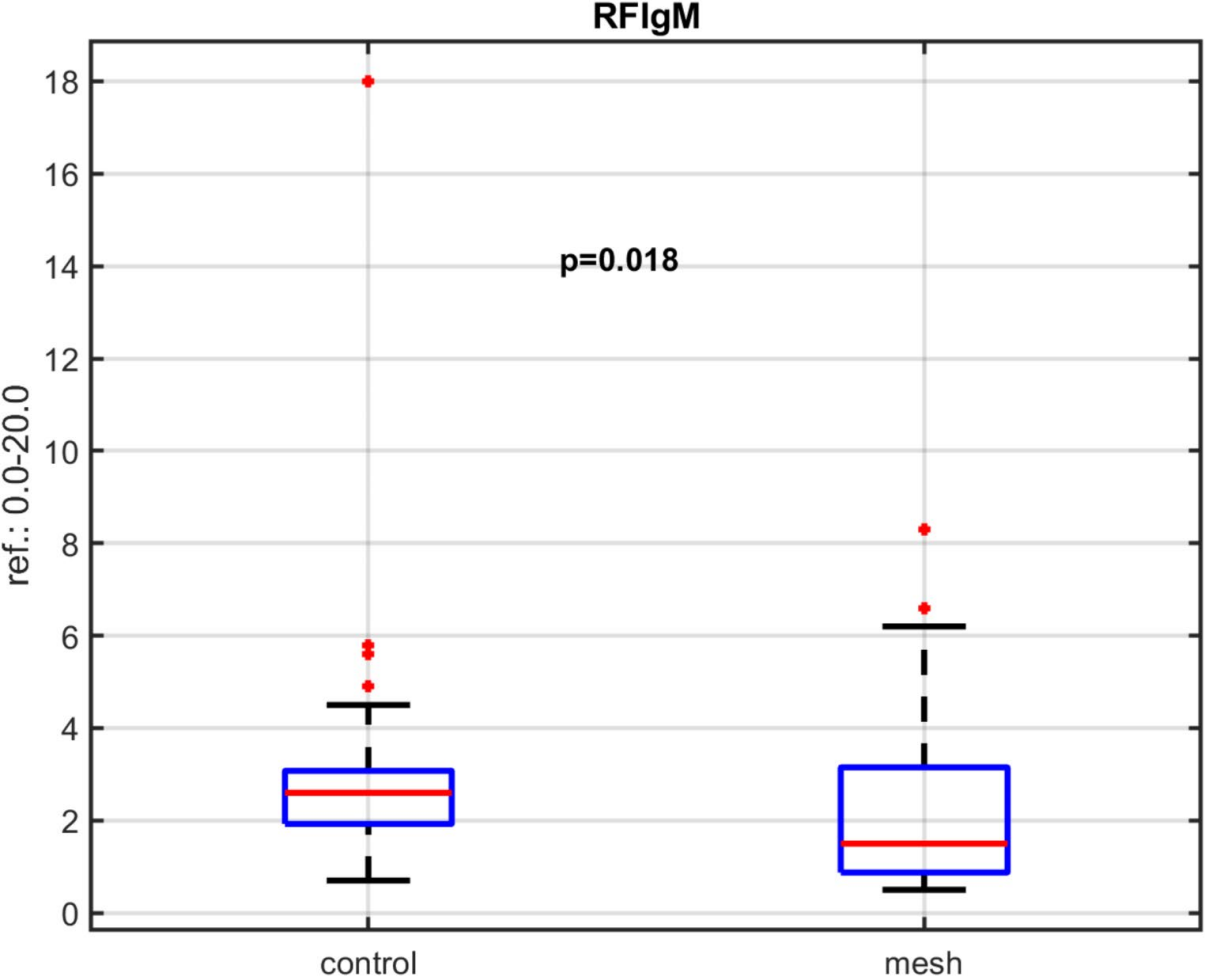
RF levels amongst patients in the mesh and control groups

A statistically significant difference in median IgG3 levels (*p* = 0.020) was observed between the group with polypropylene mesh and the control group. Despite this difference, IgG3 levels remained within the normal reference range. A statistically significant difference in median Rheumatoid Factor (RF) IgM levels (*p* = 0.018) was also observed between the group with polypropylene mesh and the control group. However, RF IgM levels remained within the normal reference range in all subjects Figs. 2 and 3.

**Fig. 4:**
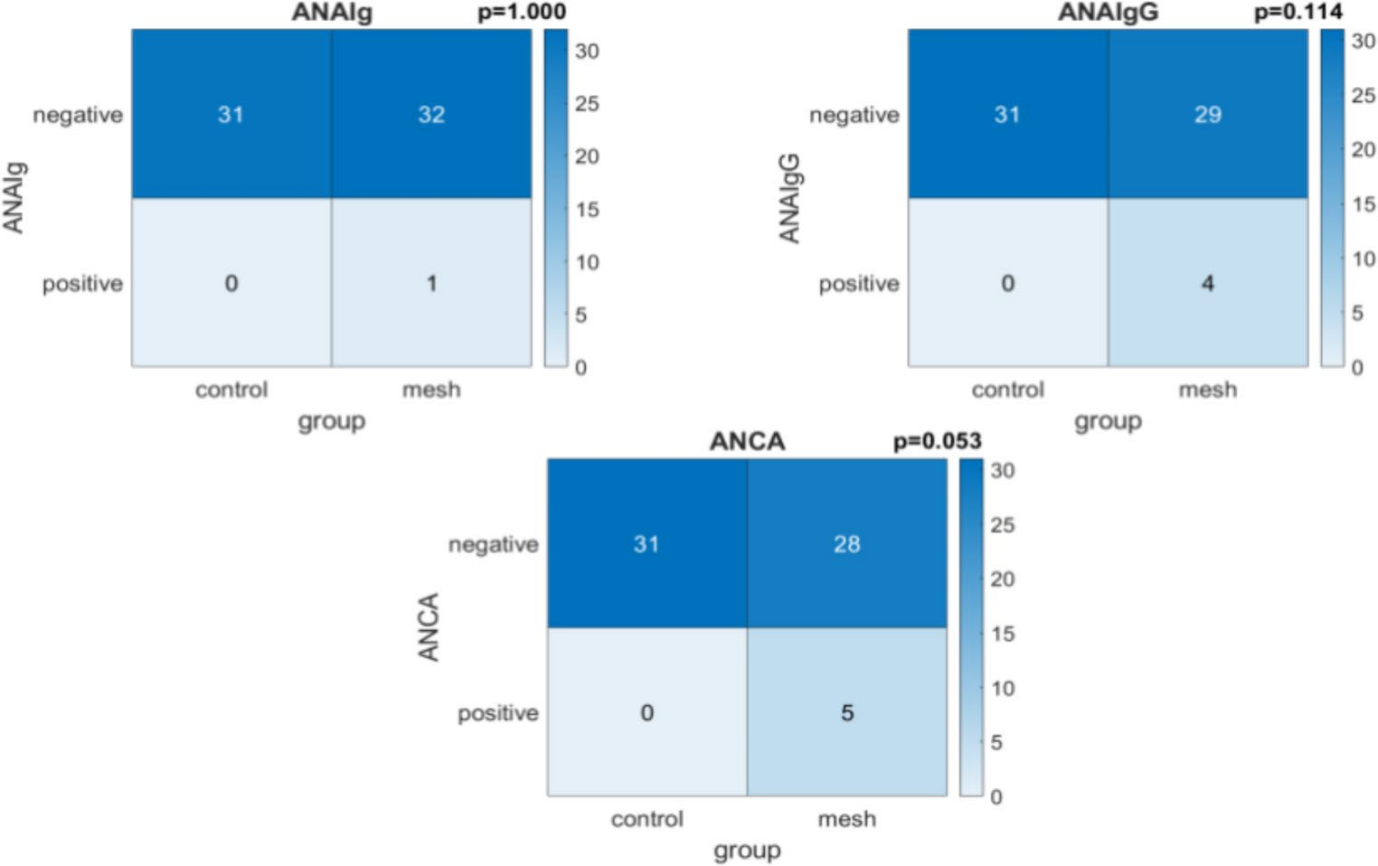
ANA and ANCA levels amongst patients in the mesh and control groups

The results for the autoantibodies findings, including antinuclear antibodies (ANA) and antineutrophil cytoplasmic antibodies (ANCA) are shown in Fig. 4. In the mesh repair group, 5 patients tested positive for ANCA compared to none in the control group, but this difference did not reach statistical significance (*p* = 0.053). One patient in the control group, who exhibited unexplained symptoms later linked to systemic lupus, tested positive for the condition and was excluded from the study, as their inclusion was inappropriate in hindsight.

Complement system component C3(*p* = 0.518) and C4(*p* = 0.704,) circulating immune complexes (CIK-C1Q: *p* = 0.463) and C - reactive protein (CRP: *p* = 0.762) were analysed by ELISA. No significant differences were observed in these parameters compared to healthy controls. Leukocyte (*p* = 0.361) and lymphocyte counts (*p* = 0.547) also showed no significant differences. Flow cytometry techniques were utilised for the enumeration of leukocytes and lymphocytes, CD3 + T cells and CD4+, CD8+, natural killer (NK) cells (CD3⁻CD16⁺CD56⁺) and B cells (CD19⁺). No significant differences were found in CD3⁺ T lymphocytes (*p* = 0.755), CD4⁺ (*p* = 0.331), CD8⁺ (*p* = 0.828), CD4⁺/CD8⁺ ratio (*p* = 0.772), NK cells (*p* = 0.242), or B cells (*p* = 0.658).

## Discussion

The present study investigated a number of serologic and cellular immunological parameters in patients a year or more after polypropylene mesh hernia repair and compared them to matched healthy controls.

Of the parameters measured, nearly all showed no differences between the two groups. The exceptions were the lower median IgG3 and RF IgM levels observed in the mesh group. Additionally, five of the mesh repair patients tested positive for ANCA.

Hernia mesh can induce a local chronic foreign body reaction that is sometimes mistaken for an allergy or autoimmunity. Both the mesh polymer and pore size significantly influence the body’s reaction to the mesh [6, 7] as well as any potential complication including hernia recurrence. The study plan was to only include patients with the same type of surgical mesh, but due to a number of implants used in the time period of the study, and many operation notes mentioning only “standard polypropylene mesh”, all patients where polypropylene mesh of any kind was used were included. In addition, there was a practice of resterilisation of mesh by autoclaving that had been opened but not used for part of the study period. Autoclaving can change the polymer surface and thus possibly influence the host-mesh interaction. This practice has now ceased. In addition, patients having a mesh hernia repair, undergo a general anaesthetic, the surgery itself as well as mesh implantation. All of these factors have the potential to initiate an immune response and thus teasing out which of these events is the most important in subsequent development of any autoimmune disease is difficult.

The present study did find that median IgG3 levels were statistically significantly lower in the mesh group compared to the control group. Nevertheless, the levels of IgG3 remained within the normal reference range in all the study subjects, IgG is the most abundant immunoglobulin in human plasma and comprises four subclasses: IgG1 (60–70%), IgG2 (20–30%), IgG3 (5–8%), and IgG4 (1–3%) [8]. The four subclasses share more than 90% structural similarity, but each subclass has distinct effector functions[9]. Although IgG3 is a minor fraction of the total IgG pool, it plays a crucial role in controlling and protecting against various pathogens. IgG3 has structural and functional heterogeneity. Often, IgG3 responses precede those of IgG1 and are characterised by potent Fc effector functions. This subclass is particularly beneficial for early pathogen responses, including intracellular antiviral activity, complement activation, antibody dependent cellular phagocytosis, and pathogen neutralisation[8–10]. Viral infections typically cause elevations in both IgG1 and IgG3, with IgG3 levels rising first[11]. Additionally, IgG1 and IgG3 are sometimes linked to autoimmune diseases, with higher or lower levels depending on the type of autoimmune disease [12]. We typically find lower levels in patients with chronic infections or pulmonary diseases (such as asthma or chronic sinusitis). The association between lower IgG3 levels and the presence of foreign materials in the body, such as surgical implants or meshes, may be attributed to several underlying immunological mechanisms[13].

Foreign materials can trigger a chronic inflammatory response as the immune system attempts to isolate or eliminate the perceived threat [14]. IgG3, known for its potent response to pathogens and large antigens, may initially play a crucial role in this process[11]. However, persistent stimulation by the foreign material may lead to dysregulation or depletion of IgG3, ultimately resulting in reduced serum levels. So in theory, chronic inflammatory states may continuously engage IgG3, leading to its consumption and subsequent decrease over time[15]. IgG3 is also particularly efficient at forming immune complexes due to its strong binding affinity for antigens. In the context of foreign materials, the immune system may produce IgG3 in response to proteins adsorbed onto the materials surface. Continuous immune complex formation and deposition could sequester IgG3 in tissues or lead to its consumption, thereby contributing to lower circulating IgG3 levels.[11].

Hypothetically, prolonged exposure to foreign materials may alter the balance of antibody production, in a way that reduces IgG3 availability [14, 16]. And it is also possible that the presence of foreign materials could potentially lead to an altered immune response, affecting the production of different antibody subclasses [14, 17]. However, the clinical relevance of this lower median in IgG3 in the cohort undergoing mesh hernia repair is unclear, especially given the fact that the median levels were lower with statistical significance, but still within the limits of the reference range. None of the mesh hernia repair patients had any symptoms to suggest the presence of autoimmune disease. With the relatively small number of patients in the study cohort, subanalysis to examine the dependence of IgG3 levels on the time since the operation was not undertaken.

The study also found that median Rheumatoid factor (RF) IgM levels were statistically significantly lower in the mesh group compared to the control group (*p* = 0.018). The levels were also within the normal reference range. Rheumatoid factor (RF) is circulating antibody in human body usually associated with rheumatoid arthritis, but it can be found in patients with other autoimmune or non-autoimmune disorders as well as in 4% of ‘healthy’ individuals [18]. RF IgM is most commonly found in the circulation[19]. RF IgM is associated with the response to a variety of antigenic stimuli including bacterial toxins, Epstein Barr virus, hepatitis C virus and chronic inflammation[18]. The observed lower median level of RF IgM in the mesh group raises the possibility of localized immune responses, which could involve the formation of immune complexes that include RF IgM. This might result in measurable RF IgM levels appearing reduced.[18, 20] However, further investigation would be necessary to determine whether these levels are actively modulated or affected by such processes. Importantly, all participants remained asymptomatic, and the median RF IgM levels in both groups were within the normal reference range.

In addition, the small sample size of this study may introduce statistical variability, which could account for the observed differences and limit the generalizability of these findings.

The present study also identified that 5 of the mesh hernia repair patients were mildly ANCA positive, compared to none in the control group. ANCA are antibodies directed against cytoplasmic antigens of neutrophils and monocytes. There are various subtypes but the most clinically relevant ANCAs target against myeloperoxidase and proteinase 3. While ANCA is most commonly associated with vasculitis, it can also be detected in other autoimmune diseases, infections, inflammatory conditions and also healthy individuals[20]. ANCA can activate neutrophils and monocytes, as well as complement systems. This activation triggers the migration of immune cells and the release of chemoattractants, leading to inflammation, apoptosis, and damage of the affected tissue[21]. ANCA can also be detected in healthy individuals in its natural form, typically at lower titres and reduced affinity to pathogens. The presence of natural ANCA in asymptomatic individuals could theoretically serve as a predictive marker for the development of autoimmune diseases. Pathogenic ANCA may arise in response to infections, pathogens, or other microbial stimuli[22]. The immune response to foreign material may involve molecular mimicry, where the immune system mistakenly targets the body’s own antigens as if they were foreign, potentially leading to the production of ANCA[23].

Any association remains speculative and has yet to be substantiated through rigorous investigation. Furthermore, ANCA positivity can be detected in 1–5% healthy individuals[24]. The diference between the groups did not reach statistical significance and none of the patients exhibited any symptoms of autoimmune disease. Although the number of affected individuals is small, this observation in the current study raises important questions regarding potential immunological responses associated with mesh repair, warranting further investigation in larger cohorts to clarify its significance and underlying mechanisms. While direct autoimmune induction by the mesh is unlikely, interactions between subclinical autoimmunity and mesh-induced inflammation are worthy of further study.

The present study has limitations, some of which have been noted above. The design of the study meant that in the mesh hernia repair group, it was not possible to measure the immune parameters prior to the surgical intervention. It is not possible to draw any definitive conclusions without knowing the pre-implantation immunological profile of our patients. The initial study population for mesh hernia repair included over 1,500 subjects. However, after applying exclusion criteria to minimize the inclusion of subjects with a high risk of, or known allergy and autoimmune disease, the final mesh hernia repair group was significantly reduced in size. This reduction underscores the selectivity of the study cohort and highlights the potential impact of sample size on the findings. Indeed, autoimmune disease is more common in the over sixties, as are hernias, adding to the scientific dilemma of what might be association versus causation. Furthermore, care was taken to carefully select the control subjects to be free from any potential autoimmune disease. But in this control group, once the immune parameter blood results were known, one control subject was diagnosed with systemic lupus and therefore was excluded as a control subject. Finally, the impact of autoclaving on the mesh surface and its potential influence on the human immune system represents another significant source of bias in this study.

The role of hernia mesh induced autoimmune disease remains a conundrum. Polypropylene and other polymer surgical meshes have become routine in the repair of millions of hernias every year worldwide for several decades now. But there has not been an obvious explosion in population-level overall incidence of autoimmunity. Nevertheless, the lower median IgG3 levels and the ANCA positivity in some patients over mesh hernia repair are intriguing and are worthy of further research.

## Conclusion

In the group of patients with polypropylene mesh, we measured lower median IgG3 and RF IgM serum levels compared to healthy controls, although within the limits of the reference range. Additionally, five patients without autoimmune-related symptoms tested mildly positive for serum ANCA. However, several other immune parameters assessed in this study showed no significant differences between the two groups. Larger prospective trials are needed to provide more comprehensive insights into this important issue.

## Declaration of generative AI and AI-assisted technologies in the writing process

During the preparation of this work the authors used ChatGPT (OpenAI) in order to improve readability and language. After using this tool, the authors reviewed and edited the content as needed and took full responsibility for the content of the publication.

## Electronic supplementary material

Below is the link to the electronic supplementary material.

Supplementary Material 1

Supplementary Material 2

Supplementary Material 3

Supplementary Material 4
