# Supplementary material for: Peripheral blood immunoprofiling in patients with polypropylene mesh implants for hernia repair: a single-center cohort study
Source: Hernia. 2025 Apr 1;29(1):131. doi: 10.1007/s10029-025-03310-1 (PMC11961455; doi:10.1007/s10029-025-03310-1)
Supplement: Supplementary file 1 — Supplementary Material 1 [file 10029_2025_3310_MOESM1_ESM.docx]

**List of abbreviations:**

EHS- European hernia society

CVD- Clavien-Dindo Classification

C3 - complement component 3

C4 - complement component 4

CD4+/CD8+ - cluster of differentiation 4/cluster of differentiation 8 ratio

ASIA - Autoimmune syndrome induced by adjuvant

(EK-361/23)- Etic Commission- reference number 361/23

MATLAB R2021b - is interactive software for engineers and scientists used for data analyses

ELISA - Enzyme-linked immunosorbent assay

IgG - immunoglobulin G

IgA - immunoglobulin A

IgM - immunoglobulin M

IgE - immunoglobulin E

CIK - circulation immunocomplex

C1Q - Circulation immunocomplex binding with 1Q

CRP- C- reactive protein

ANA - antinuclear antibodies

ANCA - Antineutrophil cytoplasmic antibodies

dsDNA - Anti-double-stranded deoxyribonucleic acid antibodies

RF - rheumatoid factor

CD3+ - cluster of differentiation 3

CD4+ - cluster of differentiation 4

CD3-16+56+ - cluster of differentiation 3-16, 56

NK cells - natural killers

CD8+ - cluster of differentiation 8

CD19+ - cluster of differentiation 19

MHC I - major histocompatibility complex I

MHC II - major histocompatibility complex II

BMI - body mass index

HIV- human immunodeficiency virus
