# Supplementary material for: Peripheral blood immunoprofiling in patients with polypropylene mesh implants for hernia repair: a single-center cohort study
Source: Hernia. 2025 Apr 1;29(1):131. doi: 10.1007/s10029-025-03310-1 (PMC11961455; doi:10.1007/s10029-025-03310-1)
Supplement: Supplementary file 2 — Supplementary Material 2 [file 10029_2025_3310_MOESM2_ESM.docx]

| Date of surgery | Blood test exam | Months after surgery | age | gender | BMI | Surgery type | IgG | IgA | IgM g | IgE | IgG1 | IgG2 | IgG3 | IgG4 | C3 | C4 | CIK C1Q | CRP | ANA Ig | ANA IgG | ANA IgA | ANA IgM | ds-DNA | ANCA | ENA+ | RF IgG | RF IgA | RF IgM | Leukocytes | Lymphocytes | Lymphocytes abs | CD3+ % | CD3+ abs | CD3- CD16+56+ | CD3- CD16+56+ abs | CD4 + | CD4+ abs | CD8+% | CD8+ abs | CD19+% | CD19+ abs | ratio CD4+/CD8+ |
| --- | --- | --- | --- | --- | --- | --- | --- | --- | --- | --- | --- | --- | --- | --- | --- | --- | --- | --- | --- | --- | --- | --- | --- | --- | --- | --- | --- | --- | --- | --- | --- | --- | --- | --- | --- | --- | --- | --- | --- | --- | --- | --- |
| 24.05.2022 | 24.05.2023 | 12 | 56 | M | 30 | umbilical | 12,7 | 2,49 | 5,66 | 46,3 | 5,99 | 5,37 | 0,531 | 0,505 | 1 | 0,2 | 79,6 | 16,6 | 0 | 0 | 0 | 0 | 0 | 0 | 4,2 | 8,7 | 2,5 | 8,3 | 5,7 | 0,34 | 2,05 | 72 | 1,52 | 17 | 0,32 | 42 | 0,85 | 25 | 0,52 | 11 | 0,22 | 1,7 |
| 27.05.2022 | 02.06.2023 | 12 | 56 | M | 29,5 | Lichtenstein | 9,57 | 0,69 | 0,51 | 4,5 | 6,27 | 2,87 | 0,229 | 0,085 | 1,03 | 0,28 | 1,2 | 3,1 | 0 | 0 | 0 | 0 | 0 | 0 | 3,8 | 2,6 | 1,5 | 4 | 6,7 | 0,41 | 1,85 | 77 | 1,12 | 11 | 0,29 | 54 | 1,66 | 16 | 0,4 | 12 | 0,16 | 3,4 |
| 23.05.2022 | 05.06.2023 | 12 | 59 | M | 24,5 | umbilical | 10,6 | 3,02 | 1,79 | 40,1 | 6,98 | 3,78 | 0,46 | 1,25 | 1,1 | 0,19 | 1,5 | 6,6 | 0 | 0 | 0 | 0 | 0 | plus | 2,8 | 5,4 | 2,9 | 3,1 | 12,7 | 0,25 | 1,52 | 74 | 0,98 | 53 | 1,55 | 20 | 0,52 | 9 | 0,33 | 9 | 0,19 | 2,7 |
| 08.11.2021 | 05.06.2023 | 15 | 41 | M | 20 | Lichtenstein | 12,6 | 2,1 | 0,89 | 59,8 | 8,22 | 2,4 | 0,38 | 2,86 | 0,8 | 0,2 | 1,1 | 3,5 | 0 | 0 | 0 | 0 | 0 | 0 | 3,2 | 4,8 | 2,2 | 1,2 | 5,9 | 0,305 | 2,38 | 75 | 1,42 | 13 | 0,4 | 50 | 0,95 | 22 | 0,45 | 12 | 0,17 | 2,3 |
| 18.11.2021 | 07.06.2023 | 15 | 51 | M | 24 | Lichtenstein | 15,1 | 1,84 | 2,59 | 13,1 | 9,44 | 6,02 | 0,724 | 1,62 | 0,71 | 0,14 | 9,7 | 1 | plus | plus | 0 | 0 | 0 | 0 | 8,1 | 7,1 | 2,4 | 3,3 | 8 | 0,288 | 2,29 | 82 | 1,23 | 8 | 0,62 | 66 | 0,66 | 14 | 0,82 | 10 | 0,05 | 4,7 |
| 23.02.2022 | 07.06.2023 | 15 | 50 | M | 23 | Lichtenstein | 10,4 | 3,38 | 1,07 | 74,9 | 4,9 | 4,89 | 0,216 | 0,537 | 0,81 | 0,21 | 1,3 | 1,3 | 0 | 0 | 0 | 0 | 0 | 0 | 3,9 | 5,7 | 3,8 | 1,7 | 5,7 | 0,35 | 2,2 | 83 | 1,68 | 6 | 0,12 | 38 | 0,77 | 40 | 0,81 | 10 | 0,2 | 1 |
| 25.01.2019 | 15.11.2023 | 46 | 45 | F | 37 | in cica | 11,9 | 2,49 | 1,54 | 28,8 | 7,03 | 6,02 | 0,47 | 0,084 | 1,15 | 0,22 | 2,1 | 8,2 | 0 | 0 | 0 | 0 | 0 | 0 | 5,7 | 4,6 | 4,4 | 3,5 | 7,6 | 0,232 | 1,76 | 70 | 1,13 | 12 | 0,21 | 46 | 0,81 | 21 | 0,37 | 15 | 0,26 | 2,2 |
| 11.11.2019 | 15.11.2023 | 48 | 44 | M | 25 | Lichtenstein | 12,3 | 2,91 | 1,38 | 41,1 | 5,47 | 5,41 | 0,452 | 1,27 | 0,96 | 0,2 | 8,7 | 3,8 | 0 | 0 | 0 | 0 | 0 | 0 | 3,7 | 7,3 | 2,3 | 1,5 | 6,4 | 0,306 | 1,96 | 56 | 1,1 | 31 | 0,61 | 25 | 0,9 | 24 | 0,47 | 8 | 0,16 | 1 |
| 13.09.2019 | 15.11.2023 | 50 | 48 | M | 29 | Lichtenstein | 12,8 | 2,85 | 0,64 | 4,8 | 7,19 | 5,28 | 0,114 | 0,974 | 1,04 | 0,38 | 1 | 5,3 | 0 | 0 | 0 | 0 | 0 | 0 | 1,6 | 4,4 | 1,8 | 6,2 | 5,4 | 0,407 | 2,2 | 61 | 1,34 | 30 | 0,66 | 28 | 0,62 | 25 | 0,55 | 7 | 0,15 | 1,1 |
| 24.09.2019 | 15.11.2023 | 50 | 48 | M | 28 | Lichtenstein | 11,3 | 1,98 | 0,87 | 61,6 | 5,38 | 5,76 | 0,561 | 0,959 | 0,86 | 0,26 | 0,9 | 2,7 | 0 | 0 | 0 | 0 | 0 | 0 | 4 | 4,8 | 1,9 | 2 | 6,2 | 0,333 | 2,06 | 59 | 1,22 | 25 | 0,52 | 33 | 0,68 | 20 | 0,41 | 11 | 0,23 | 1,7 |
| 03.12.2018 | 15.11.2023 | 47 | 46 | F | 23 | Lichtenstein | 9,92 | 1,1 | 1,15 | 53,2 | 6,32 | 7,74 | 0,706 | 0,634 | 0,78 | 0,2 | 1,3 | 1,6 | 0 | 0 | 0 | 0 | 0 | 0 | 2,1 | 5,9 | 1,6 | 1,6 | 5,9 | 0,411 | 2,42 | 74 | 1,79 | 15 | 0,36 | 48 | 1,16 | 19 | 0,46 | 7 | 0,17 | 2,5 |
| 10.02.2020 | 15.11.2023 | 45 | 27 | M | 20 | Lichtenstein | 13,2 | 3,04 | 0,72 | 7,7 | 6,44 | 5,76 | 0,361 | 1,52 | 0,78 | 0,15 | 100 | 1 | 0 | 0 | 0 | 0 | 0 | 0 | 6,5 | 6,6 | 1,8 | 1,1 | 7,3 | 0,413 | 3,01 | 38 | 1,14 | 56 | 1,68 | 22 | 0,66 | 13 | 0,39 | 3 | 0,08 | 1,7 |
| 06.11.2019 | 15.11.2023 | 48 | 58 | F | 26,4 | in cica | 8,73 | 0,97 | 0,66 | 10,3 | 5,24 | 3,02 | 0,214 | 0,175 | 1,02 | 0,22 | 0,7 | 1,1 | 0 | 0 | 0 | 0 | 0 | 0 | 1,9 | 2,4 | 1,3 | 3,3 | 6,6 | 0,32 | 2,11 | 72 | 1,52 | 16 | 0,34 | 30 | 0,63 | 37 | 0,78 | 8 | 0,17 | 0,8 |
| 21.02.2019 | 15.11.2023 | 57 | 54 | M | 26 | Lichtenstein | 11,7 | 3,21 | 0,72 | 4,1 | 7,16 | 3,04 | 0,139 | 1,51 | 1,09 | 0,19 | 1,2 | 1,4 | 0 | 0 | 0 | 0 | 0 | 0 | 3,7 | 8 | 3,6 | 6,6 | 5,8 | 0,398 | 2,31 | 72 | 1,66 | 18 | 0,42 | 39 | 0,9 | 29 | 0,67 | 8 | 0,18 | 1,3 |
| 04.09.2018 | 15.11.2023 | 62 | 43 | F | 26 | Lichtenstein | 12,7 | 1,99 | 2,52 | 19,2 | 7,23 | 3,75 | 0,489 | 0,125 | 0,83 | 0,29 | 3,3 | 2,5 | 0 | 0 | 0 | 0 | 0 | plus | 5,2 | 3,1 | 1,8 | 4 | 5,7 | 0,264 | 1,5 | 74 | 1,11 | 16 | 0,24 | 40 | 0,6 | 23 | 0,35 | 7 | 0,11 | 1,7 |
| 01.04.2019 | 15.11.2023 | 54 | 34 | M | 27 | Lichtenstein | 9,49 | 3,04 | 0,61 | 5,5 | 5,18 | 4,13 | 0,441 | 0,9 | 1,18 | 0,23 | 56,2 | 93 | 0 | 0 | 0 | 0 | 0 | 0 | 2,4 | 4,6 | 1,7 | 0,7 | 5,9 | 0,187 | 1,1 | 66 | 0,73 | 17 | 0,19 | 46 | 0,51 | 15 | 0,17 | 11 | 0,12 | 3,1 |
| 08.10.2019 | 16.11.2023 | 49 | 50 | M | 25 | Lichtenstein | 8,87 | 2,78 | 0,78 | 10,7 | 6 | 3,4 | 0,406 | 0,205 | 0,65 | 0,1 | 0,7 | 1,9 | 0 | 0 | 0 | 0 | 0 | 0 | 3,7 | 2,8 | 1,5 | 0,5 | 4,2 | 0,354 | 1,49 | 75 | 1,12 | 18 | 0,27 | 47 | 0,7 | 20 | 0,3 | 3 | 0,04 | 2,4 |
| 22.11.2022 | 16.11.2023 | 12 | 52 | M | 26 | Lichtenstein | 13,7 | 3,78 | 1,94 | 17,8 | 6,97 | 7,48 | 0,616 | 1,31 | 0,92 | 0,19 | 3 | 9,8 | 0 | 0 | 0 | 0 | 0 | plus | 3 | 8,1 | 4,6 | 1 | 5,4 | 0,363 | 1,96 | 76 | 1,49 | 19 | 0,37 | 48 | 0,94 | 26 | 0,51 | 3 | 0,06 | 1,8 |
| 15.10.2019 | 16.11.2023 | 49 | 50 | F | 22,5 | in cica | 9,24 | 1,57 | 0,9 | 1 | 5,2 | 4,1 | 0,208 | 0,993 | 0,96 | 0,15 | 3,5 | 2,3 | plus | plus | 0 | 0 | 0 | 0 | 4,5 | 5,8 | 1,3 | 1,3 | 5,9 | 0,257 | 1,52 | 65 | 0,99 | 19 | 0,29 | 51 | 0,78 | 12 | 0,18 | 12 | 0,18 | 4,3 |
| 10.02.2020 | 16.11.2023 | 45 | 59 | M | 27 | Lichtenstein | 10,7 | 1,88 | 0,87 | 94,6 | 6,93 | 3,78 | 0,259 | 1,32 | 0,85 | 0,19 | 0,8 | 1,7 | 0 | 0 | 0 | 0 | 0 | 0 | 4,5 | 7,7 | 1,3 | 1,3 | 7 | 0,314 | 2,2 | 72 | 1,58 | 16 | 0,35 | 43 | 0,95 | 23 | 0,51 | 8 | 0,18 | 1,9 |
| 08.01.2020 | 16.11.2023 | 46 | 54 | F | 27 | in cica | 10,2 | 1,75 | 0,95 | 4 | 5,99 | 3,93 | 0,172 | 0,084 | 1,02 | 0,17 | 1 | 2,4 | 0 | 0 | 0 | 0 | 0 | 0 | 2,2 | 1,5 | 7,9 | 0,7 | 10,9 | 0,317 | 3,4 | 77 | 2,66 | 15 | 0,52 | 45 | 1,56 | 28 | 0,97 | 6 | 0,21 | 01.VI |
| 22.11.2018 | 16.11.2023 | 60 | 59 | M | 29,4 | in cica | 8,47 | 1,17 | 0,17 | 1,5 | 5,85 | 2,62 | 0,295 | 0,222 | 1,39 | 0,21 | 1,3 | 3,2 | 0 | 0 | 0 | 0 | 0 | 0 | 2,5 | 3,1 | 1,4 | 0,5 | 7,5 | 0,398 | 2,99 | 69 | 2,06 | 25 | 0,75 | 44 | 1,32 | 20 | 0,6 | 4 | 0,12 | 2,2 |
| 09.02.2018 | 16.11.2023 | 69 | 39 | M | 26,8 | Lichtenstein | 9,26 | 4,75 | 0,32 | 98,4 | 5,79 | 3,45 | 0,26 | 0,376 | 0,88 | 0,14 | 0,8 | 5,5 | 0 | 0 | 0 | 0 | 0 | 0 | 1,8 | 4,4 | 3,2 | 0,8 | 7,9 | 0,411 | 3,25 | 82 | 2,67 | 3 | 0,1 | 51 | 1,66 | 27 | 0,88 | 14 | 0,46 | 1,9 |
| 29.01.2019 | 19.11.2023 | 58 | 39 | M | 27 | Lichtenstein | 7,5 | 2,18 | 1,45 | 83,5 | 7,51 | 5,43 | 0,371 | 2,16 | 0,89 | 0,23 | 2,5 | 2,6 | 0 | 0 | 0 | 0 | 0 | 0 | 2,5 | 7,9 | 1,3 | 1,2 | 8,1 | 0,191 | 1,55 | 64 | 0,99 | 23 | 0,36 | 38 | 0,59 | 23 | 0,36 | 10 | 0,16 | 1,7 |
| 02.12.2019 | 10.11.2023 | 47 | 36 | F | 23 | in cica | 15,6 | 1,86 | 2,75 | 38,4 | 10,1 | 5,08 | 0,457 | 1,1 | 0,71 | 0,11 | 2,3 | 1,8 | plus | plus | 0 | 0 | 0 | 0 | 1,8 | 3,2 | 1,6 | 0,8 | 7,9 | 0,401 | 3,2 | 81 | 2,1 | 9% | 0,12 | 32 | 1,65 | 45 | 0,45 | 7 | 0,12 | 0,7 |
| 02.05.2019 | 13.11.2023 | 55 | 54 | M | 24,6 | in cica | 9,23 | 1,27 | 0,59 | 160 | 5,03 | 2,31 | 0,23 | 1,6 | 0,92 | 0,34 | 0,9 | 3,5 | 0 | 0 | 0 | 0 | 0 | 0 | 1,4 | 10 | 2,1 | 1,6 | 12 | 0,211 | 2,53 | 73 | 1,85 | 12 | 0,3 | 59 | 1,49 | 12 | 0,3 | 8 | 0,2 | 4,9 |
| 21.06.2018 | 13.11.2023 | 65 | 47 | M | 29,4 | Lichtenstein | 8,12 | 1,75 | 0,45 | 40,3 | 4,29 | 3,02 | 0,306 | 0,764 | 0,79 | 0,15 | 0,9 | 1,5 | 0 | 0 | 0 | 0 | 0 | 0 | 1,2 | 3,8 | 1,3 | 0,6 | 3,5 | 0,31 | 1,1 | 63 | 0,69 | 20 | 0,22 | 43 | 0,47 | 16 | 0,18 | 10 | 0,11 | 2,7 |
| 10.05.2018 | 10.11.2023 | 66 | 43 | M | 29,9 | Lichtenstein | 9,02 | 1,36 | 0,82 | 8,1 | 7,08 | 2,45 | 0,618 | 0,267 | 0,8 | 0,16 | 1,2 | 1,7 | 0 | 0 | 0 | 0 | 0 | plus | 2,6 | 4,1 | 1,3 | 2,8 | 6,3 | 0,35 | 2,21 | 66 | 1,46 | 25 | 0,55 | 39 | 0,86 | 20 | 0,44 | 5 | 0,11 | 2 |
| 22.01.2018 | 14.11.2023 | 70 | 48 | M | 39 | Lichtenstein | 5,45 | 0,76 | 0,35 | 34,2 | 3,04 | 1,95 | 0,272 | 0,376 | 0,98 | 0,2 | 13,8 | 1,9 | 0 | 0 | 0 | 0 | 0 | 0 | 1,3 | 5,3 | 1,9 | 0,9 | 11 | 0,283 | 3,11 | 72 | 2,24 | 8 | 0,25 | 50 | 1,56 | 19 | 0,59 | 16 | 0,5 | 2,6 |
| 27.09.2018 | 22.11.2023 | 62 | 51 | M | 25 | Lichtenstein | 10,5 | 1,87 | 1,54 | 111 | 5,5 | 3,99 | 0,173 | 0,741 | 0,88 | 0,21 | 1,4 | 3,8 | 0 | 0 | 0 | 0 | 0 | plus | 5,1 | 5,5 | 2,5 | 1,7 | 6,8 | 0,421 | 2,86 | 80 | 2,29 | 14 | 0,4 | 38 | 1,09 | 37 | 1,06 | 4 | 0,11 | 1 |
| 29.01.2019 | 19.11.2023 | 58 | 39 | M | 27 | Lichtenstein | 10,2 | 2,18 | 1,45 | 83,5 | 7,51 | 5,43 | 0,371 | 2,16 | 0,89 | 0,23 | 2,5 | 2,6 | 0 | 0 | 0 | 0 | 0 | 0 | 2,5 | 7,9 | 1,3 | 1,2 | 8,1 | 0,191 | 1,55 | 64 | 0,99 | 23 | 0,36 | 38 | 0,59 | 23 | 0,36 | 10 | 0,16 | 1,7 |
| 26.01.2018 | 25.11.2023 | 58 | 41 | M | 29,8 | Lichtenstein | 11 | 0,65 | 1,06 | 148 | 7,88 | 2,27 | 0,391 | 0,472 | 1,11 | 0,21 | 1 | 7,5 | plus | plus | 0 | 0 | 0 | 0 | 2,2 | 4,2 | 1,3 | 1,5 | 2,1 | 0,293 | 2,1 | 62 | 1,1 | 15 | 0,31 | 40 | 0,75 | 20 | 0,44 | 10 | 0,16 | 2,1 |
| 12.06.2019 | 07.12.2023 | 54 | 49 | M | 29,6 | Lichtenstein | 14,9 | 2,05 | 0,47 | 171 | 8,07 | 6,99 | 0,876 | 1,66 | 0,83 | 0,16 | 17,3 | 1,4 | 0 | 0 | 0 | 0 | 0 | 0 | 7,8 | 5,6 | 1,9 | 0,7 | 5,6 | 0,413 | 2,31 | 67 | 1,55 | 22 | 0,51 | 46 | 1,06 | 15 | 0,35 | 9 | 0,21 | 3,1 |
