# Supplementary material for: Peripheral blood immunoprofiling in patients with polypropylene mesh implants for hernia repair: a single-center cohort study
Source: Hernia. 2025 Apr 1;29(1):131. doi: 10.1007/s10029-025-03310-1 (PMC11961455; doi:10.1007/s10029-025-03310-1)
Supplement: Supplementary file 3 — Supplementary Material 3 [file 10029_2025_3310_MOESM3_ESM.docx]

| blood test | BMI | Age | Gender | IgG | IgA | IgM g | IgE | IgG1 | IgG2 | IgG3 | IgG4 | C3 | C4 | CIK C1Q | CRP | ANA Ig | ANA IgG | ANA IgA | ANA IgM | ds- DNA | ANCA | ENA+ | RF IgG | RF IgA | RF IgM | Leucocytes | Lymphocytes | Lymphoc. abs | CD3+% | CD3+ abs | CD3- CD16+56+ % | CD3 -CD16+56+ abs | CD4 + | CD4+ abs | CD8+% | CD8+ abs | CD19+ % | CD19+ abs | ratio CD4+/CD8+ |
| --- | --- | --- | --- | --- | --- | --- | --- | --- | --- | --- | --- | --- | --- | --- | --- | --- | --- | --- | --- | --- | --- | --- | --- | --- | --- | --- | --- | --- | --- | --- | --- | --- | --- | --- | --- | --- | --- | --- | --- |
| 25.05.2023 | 22,5 | 51 | F | 10,8 | 0,87 | 1,35 | 95 | 5,15 | 5,82 | 0,682 | 0,07 | 0,7 | 0,13 | 19,1 | 1,7 | 0 | 0 | 0 | 0 | 0 | 0 | 1,2 | 2,5 | 1,9 | 2,6 | 4,7 | 0,25 | 1,52 | 71 | 0,98 | 18 | 0,43 | 55 | 0,82 | 12 | 0,55 | 11 | 0,33 | 4,6 |
| 25.05.2023 | 18,5 | 43 | F | 12,4 | 2,55 | 0,64 | 42,2 | 8,41 | 3,07 | 0,366 | 0,905 | 0,69 | 0,14 | 1,1 | 1,1 | 0 | 0 | 0 | 0 | 0 | 0 | 2,7 | 6,8 | 1,8 | 0,9 | 4 | 0,412 | 1,58 | 78 | 1,15 | 12 | 0,71 | 55 | 1,2 | 21 | 0,31 | 10 | 0,08 | 2,6 |
| 24.10.2023 | 24,4 | 31 | M | 11,8 | 2,51 | 1,18 | 76,1 | 7,1 | 4,11 | 0,589 | 0,92 | 0,72 | 0,15 | 10,6 | 1,6 | 0 | 0 | 0 | 0 | 0 | 0 | 11,2 | 10,6 | 3,1 | 4,5 | 5,7 | 0,295 | 1,68 | 68 | 1,14 | 19 | 0,32 | 40 | 0,67 | 21 | 0,34 | 7 | 0,12 | 1,9 |
| 15.11.2023 | 18,5 | 30 | F | 12 | 1,78 | 1,16 | 24 | 7,01 | 5,2 | 0,346 | 1,32 | 0,7 | 0,15 | 5,6 | 1,4 | 0 | 0 | 0 | 0 | 0 | 0 | 5 | 6,7 | 1,4 | 2,2 | 4,8 | 0,33 | 1,6 | 73 | 1,17 | 14 | 0,22 | 49 | 0,78 | 21 | 0,34 | 10 | 0,16 | 2,3 |
| 15.11.2023 | 21 | 59 | F | 9,19 | 2,52 | 1,84 | 140 | 4,58 | 4,59 | 0,33 | 0,275 | 0,9 | 0,21 | 0,9 | 1,8 | 0 | 0 | 0 | 0 | 0 | 0 | 1,4 | 4,2 | 1,9 | 2,4 | 5,2 | 0,379 | 1,97 | 85 | 1,67 | 7 | 0,14 | 53 | 1,04 | 29 | 0,57 | 7 | 0,14 | 1,8 |
| 15.11.2023 | 20 | 39 | M | 9,7 | 4,44 | 0,42 | 28,2 | 6,55 | 3,89 | 0,867 | 0,208 | 0,91 | 0,26 | 3,2 | 2 | 0 | 0 | 0 | 0 | 0 | 0 | 2,9 | 3,6 | 2,5 | 3,1 | 4,3 | 0,339 | 1,46 | 67 | 0,98 | 25 | 0,37 | 45 | 0,66 | 17 | 0,25 | 6 | 0,06 | 2,6 |
| 15.11.2023 | 25 | 52 | M | 12,2 | 2,15 | 0,96 | 12,1 | 7,22 | 3,44 | 0,405 | 2,25 | 0,75 | 0,17 | 2,1 | 1,7 | 0 | 0 | 0 | 0 | 0 | 0 | 2 | 6,5 | 1,9 | 0,7 | 5,4 | 0,346 | 1,87 | 67 | 1,25 | 17 | 0,32 | 56 | 1,05 | 7 | 0,13 | 13 | 0,24 | 8 |
| 15.11.2023 | 25,5 | 23 | M | 11 | 1,77 | 0,66 | 15,5 | 6,32 | 4,26 | 0,703 | 0,885 | 0,81 | 0,16 | 9,3 | 1,5 | 0 | 0 | 0 | 0 | 0 | 0 | 5,8 | 7,7 | 1,8 | 1,6 | 5,9 | 0,407 | 2,4 | 68 | 1,63 | 17 | 0,41 | 35 | 0,84 | 21 | 0,5 | 11 | 0,26 | 1,7 |
| 08.07.2024 | 23,4 | 47 | M | 9,51 | 1,81 | 1,42 | 119 | 6,7 | 2,56 | 0,659 | 0,552 | 1,19 | 0,34 | 0,8 | 7,5 | 0 | 0 | 0 | 0 | 0 | 0 | 3,6 | 9,5 | 2,3 | 1,9 | 5,9 | 0,337 | 1,99 | 74 | 1,47 | 13 | 0,26 | 50 | 1 | 21 | 0,42 | 7 | 0,14 | 2,4 |
| 08.07.2024 | 22,5 | 51 | M | 11 | 0,74 | 0,55 | 16,1 | 6,63 | 4 | 0,319 | 0,807 | 1,09 | 0,19 | 0,5 | 4,7 | 0 | 0 | 0 | 0 | 0 | 0 | 3,6 | 5,1 | 1,3 | 2 | 5,7 | 0,329 | 1,88 | 69 | 1,3 | 18 | 0,34 | 47 | 0,88 | 18 | 0,34 | 11 | 0,21 | 2,6 |
| 08.07.2024 | 23,4 | 47 | M | 14,4 | 1,85 | 1,58 | 3,8 | 7,59 | 7,26 | 0,491 | 0,733 | 1,06 | 0,25 | 2,5 | 4,7 | 0 | 0 | 0 | 0 | 0 | 0 | < 3,6 | 5,9 | 19,3 | 4,9 | 5,5 | 0,401 | 2,21 | 60 | 1,33 | 26 | 0,57 | 30 | 0,66 | 23 | 0,51 | 10 | 0,22 | 1,3 |
| 08.07.2024 | 21,1 | 42 | M | 11,9 | 1,69 | 1,09 | 8,3 | 7,9 | 3,58 | 1,06 | 0,215 | 0,95 | 0,24 | 2,1 | 2,7 | 0 | 0 | 0 | 0 | 0 | 0 | < 3,6 | 3,7 | 2,2 | 2,5 | 5,3 | 0,35 | 1,86 | 65 | 1,21 | 15 | 0,28 | 47 | 0,87 | 16 | 0,3 | 15 | 0,28 | 2,9 |
| 08.07.2024 | 25,2 | 48 | M | 11,8 | 2,75 | 0,63 | 20,3 | 9,24 | 3,5 | 0,59 | 0,88 | 0,92 | 0,19 | 2,4 | 2,6 | 0 | 0 | 0 | 0 | 0 | 0 | <3,6 | 5 | 2 | 3,5 | 8,5 | 0,358 | 3,04 | 74 | 2,25 | 17 | 0,52 | 25 | 0,76 | 35 | 1,06 | 4,8 | 0,15 | 0,7 |
| 08.07.2024 | 26,3 | 47 | M | 9,01 | 0,78 | 1,92 | 12,8 | 6,42 | 3,42 | 0,527 | 0,225 | 0,88 | 0,18 | 4,7 | 1,6 | 0 | 0 | 0 | 0 | 0 | 0 | <3,6 | 4,7 | 2,1 | 5,6 | 5,9 | 0,31 | 2,83 | 84 | 1,54 | 9 | 0,16 | 50 | 0,92 | 30 | 0,55 | 3,7 | 0,07 | 1,7 |
| 08.07.2024 | 25,4 | 46 | M | 12,6 | 3,51 | 1,61 | 250 | 6,44 | 3,43 | 0,801 | 2,025 | 1,08 | 0,21 | 3,1 | 5 | 0 | 0 | 0 | 0 | 0 | 0 | <3,6 | 7,3 | 3,3 | 3,4 | 6,9 | 0,38 | 2,62 | 77 | 2,02 | 7 | 0,18 | 40 | 1,05 | 31 | 0,81 | 12 | 0,31 | 1,3 |
| 09.07.2024 | 21 | 53 | M | 10,4 | 2,54 | 0,76 | 90,9 | 5,45 | 4,79 | 0,318 | 0,746 | 0,83 | 0,19 | 2,1 | 1,4 | 0 | 0 | 0 | 0 | 0 | 0 | 4,8 | 4 | 1,7 | 2 | 6,9 | 0,262 | 1,81 | 69 | 1,25 | 23 | 0,42 | 34 | 0,62 | 30 | 0,54 | 5 | 0,09 | 1,1 |
| 09.07.2024 | 26,5 | 43 | M | 5,88 | 1,01 | 1,07 | 7,1 | 4,28 | 1,64 | 0,354 | 0,482 | 0,99 | 0,14 | 0,7 | 2,6 | 0 | 0 | 0 | 0 | 0 | 0 | <3,6 | 4,6 | 1,8 | 2,5 | 10,5 | 0,349 | 3,66 | 79 | 2,89 | 9 | 0,33 | 58 | 2,12 | 17 | 0,62 | 6 | 0,22 | 3,4 |
| 11.07.2024 | 24 | 46 | M | 13,8 | 2,66 | 0,61 | 89,9 | 7,1 | 5,51 | 0,613 | 5,595 | 1,25 | 0,28 | 0,7 | 4,9 | 0 | 0 | 0 | 0 | 0 | 0 | <3,6 | 4,7 | 1,5 | 18 | 5,5 | 0,399 | 2,19 | 71 | 1,55 | 22 | 0,48 | 48 | 1,05 | 21 | 0,46 | 4 | 0,09 | 2,3 |
| 11.07.2024 | 23 | 44 | M | 11,9 | 3,06 | 0,65 | 12 | 9,79 | 4,7 | 0,902 | 1,4 | 1,29 | 0,3 | 0,7 | 4,4 | 0 | 0 | 0 | 0 | 0 | 0 | <3,6 | 6,3 | 2,3 | 2,8 | 6,7 | 0,33 | 2,21 | 67 | 1,48 | 20 | 0,44 | 41 | 0,91 | 16 | 0,35 | 9 | 0,2 | 2,6 |
| 16.07.2024 | 28,2 | 46 | M | 9,1 | 12 | 9,79 | 47,4 | 4,87 | 4,67 | 0,436 | 1,987 | 0,86 | 0,22 | 1,3 | 6,3 | 0 | 0 | 0 | 0 | 0 | 0 | <3,6 | 5 | 1,4 | 2,8 | 8,3 | 0,086 | 0,71 | 63 | 0,45 | 12 | 0,009 | 37 | 0,26 | 23 | 0,16 | 19 | 0,13 | 1,6 |
| 16.07.2024 | 24 | 49 | M | 12,4 | 2,94 | 1,56 | 119 | 9,24 | 3,94 | 0,584 | 0,821 | 0,96 | 0,25 | 0,7 | 2,8 | 0 | 0 | 0 | 0 | 0 | 0 | <3,6 | 5,3 | 1,9 | 3 | 8,5 | 0,269 | 2,29 | 64 | 1,47 | 26 | 0,6 | 37 | 0,85 | 21 | 0,48 | 9 | 0,21 | 1,8 |
| 10.07.2024 | 23,1 | 40 | M | 12 | 1,41 | 0,38 | 85,7 | 7,38 | 3,97 | 0,694 | 0,751 | 0,98 | 0,33 | 3,5 | 5,1 | 0 | 0 | 0 | 0 | 0 | 0 | 6,3 | 5 | 2,1 | 1 | 3,1 | 0,502 | 1,56 | 71 | 1,11 | 19 | 0,3 | 40 | 0,62 | 28 | 0,44 | 6 | 0,09 | 1,4 |
| 10.07.2024 | 21,1 | 51 | M | 9,79 | 1,25 | 0,7 | 22,6 | 5,98 | 3,55 | 0,418 | 0,27 | 1,05 | 0,17 | 0,8 | 2,4 | 0 | 0 | 0 | 0 | 0 | 0 | <3,6 | 3,9 | 2,1 | 1,1 | 5,5 | 0,42 | 2,1 | 73 | 1,52 | 19 | 0,32 | 54 | 0,92 | 15 | 0,72 | 3 | 0,23 | 3,6 |
| 16.07.2024 | 25,2 | 44 | M | 11 | 3,74 | 0,8 | 91 | 7,22 | 4,7 | 0,672 | 0,89 | 0,86 | 0,1 | 1 | 1,7 | 0 | 0 | 0 | 0 | 0 | 0 | <3,6 | 6,5 | 3,1 | 2,8 | 7,1 | 0,387 | 2,75 | 62 | 1,71 | 22 | 0,61 | 33 | 0,91 | 26 | 0,72 | 10 | 0,28 | 1,3 |
| 16.07.2024 | 25,5 | 47 | M | 13,2 | 4,42 | 0,98 | 111 | 8,96 | 4,71 | 0,236 | 1,892 | 1,12 | 0,23 | 1 | 2,5 | 0 | 0 | 0 | 0 | 0 | 0 | <3,6 | 6,8 | 2,6 | 2,8 | 9,3 | 0,372 | 3,46 | 62 | 2,15 | 23 | 0,8 | 43 | 1,49 | 18 | 0,62 | 12 | 0,42 | 2,4 |
| 09.07.2024 | 25,7 | 58 | F | 12,9 | 1,77 | 0,86 | 11,3 | 8,31 | 3,75 | 0,599 | 1,05 | 1,36 | 0,17 | 1,1 | 2,7 | 0 | 0 | 0 | 0 | 0 | 0 | <3,6 | 4,9 | 2,3 | 1,2 | 6,6 | 0,172 | 1,14 | 70 | 0,8 | 14 | 0,16 | 47 | 0,54 | 19 | 0,22 | 14 | 0,16 | 2,5 |
| 15.07.2024 | 28,5 | 50 | F | 7,58 | 0,61 | 1,27 | 15,3 | 4,8 | 2,84 | 0,2 | 0,095 | 1,35 | 0,33 | 1,3 | 4,3 | 0 | 0 | 0 | 0 | 0 | 0 | 6,1 | 2,4 | 0,8 | 2,8 | 10,4 | 0,315 | 3,28 | 79 | 2,59 | 11 | 0,36 | 53 | 1,74 | 24 | 0,79 | 7 | 0,23 | 2,2 |
| 09.07.2024 | 20,8 | 42 | M | 11,2 | 2,44 | 0,79 | 881 | 6,55 | 2,56 | 0,294 | 3,3 | 0,9 | 0,35 | 0,9 | 2,4 | 0 | 0 | 0 | 0 | 0 | 0 | <3,6 | 5,7 | 1,5 | 1,8 | 7,3 | 0,373 | 2,72 | 76 | 2,07 | 10 | 0,27 | 48 | 1,33 | 26 | 0,71 | 10 | 0,27 | 1,8 |
| 16.07.2024 | 21 | 41 | M | 10,6 | 1,67 | 0,71 | 65,5 | 8,55 | 3,85 | 0,77 | 1,9 | 0,65 | 0,18 | 0,7 | 1,4 | 0 | 0 | 0 | 0 | 0 | 0 | <3,6 | 6,4 | 1,4 | 5,8 | 6,4 | 0,27 | 1,73 | 61 | 1,06 | 20 | 0,35 | 30 | 0,52 | 27 | 0,47 | 11 | 0,19 | 1,1 |
| 10.07.2024 | 26,1 | 44 | M | **9,77** | 1,96 | 0,97 | 25,9 | 5,91 | 2,23 | 0,322 | 0,86 | 1,15 | 0,28 | 4,1 | 5,3 | 0 | 0 | 0 | 0 | 0 | 0 | <3,6 | 5,5 | 1,8 | 2,3 | 6,2 | 0,28 | 1,74 | 74 | 1,29 | 9 | 0,16 | 48 | 0,84 | 23 | 0,4 | 11 | 0,19 | 2,1 |
| 11.07.2024 | 25,5 | 44 | M | 12,6 | 2,05 | 2,19 | 71,9 | 8,23 | 3,29 | 1,11 | 0,84 | 0,71 | 0,014 | 12,7 | 1,7 | 0 | 0 | 0 | 0 | 0 | 0 | <3,6 | 5,2 | 1,8 | 2,7 | 6,2 | 0,389 | 2,41 | 71 | 1,71 | 17 | 0,41 | 39 | 0,94 | 24 | 0,58 | 8 | 0,19 | 1,6 |
