# Supplementary material for: Peripheral blood immunoprofiling in patients with polypropylene mesh implants for hernia repair: a single-center cohort study
Source: Hernia. 2025 Apr 1;29(1):131. doi: 10.1007/s10029-025-03310-1 (PMC11961455; doi:10.1007/s10029-025-03310-1)
Supplement: Supplementary file 4 — Supplementary Material 4 [file 10029_2025_3310_MOESM4_ESM.docx]

**Conflict of interests**

The authors declare that the research was conducted in the absence of any commercial or financial relationships and there is no conflict of interest.

This research was supported by a research grant from EHS.
